# CEUS and CT/MRI LI-RADS in Association With Serum Biomarkers for Differentiation of Combined Hepatocellular-Cholangiocarcinoma From Hepatocellular Carcinoma

**DOI:** 10.3389/fonc.2022.897090

**Published:** 2022-05-16

**Authors:** Yan Zhou, Shanshan Yin, Lin Zhao, Xiang Zhang, Meng Li, Jianmin Ding, Kun Yan, Xiang Jing

**Affiliations:** ^1^ School of Medicine, Nankai University, Tianjin, China; ^2^ Department of Ultrasound, Tianjin Third Central Hospital, Tianjin, China; ^3^ Tianjin Institute of Hepatobiliary Disease, Tianjin Key Laboratory of Extracorporeal Life Support for Critical Diseases, Artificial Cell Engineering Technology Research Center, Tianjin Third Central Hospital, Tianjin, China; ^4^ Key Laboratory of Carcinogenesis and Translational Research (Ministry of Education/Beijing), Department of Ultrasound, Peking University Cancer Hospital and Institute, Beijing, China; ^5^ Department of Radiology, Tianjin Third Central Hospital, Tianjin, China

**Keywords:** liver imaging reporting and data system, contrast-enhanced ultrasound, contrast-enhanced magnetic resonance imaging, contrast-enhanced computed tomography, combined hepatocellular-cholangiocarcinoma

## Abstract

**Background:**

Combined Hepatocellular-cholangiocarcinoma (cHCC-CCAs) are with both unambiguously differentiated hepatocellular and biliary components. cHCC-CCAs show various imaging features similar to hepatocellular carcinoma (HCCs) and intrahepatic cholangiocarcinoma (ICCs), which makes the differential diagnosis between them challenging. The accurate diagnosis of cHCC-CCAs is of great importance in selecting treatment methods and performing patient management.

**Purpose:**

To investigate the diagnostic efficacy of CEUS and CT/MRI LI-RADS in association with tumor biomarkers for differentiation of cHCC-CCAs from HCCs.

**Methods:**

A total of 54 cHCC-CCAs and 55 HCCs in two centers were retrospectively collected. The diagnostic criteria for cHCC-CCAs if one or more of the following conditions were satisfied: (1) arterial phase hyperenhancement (APHE) on CEUS and LR-M on CT/MRI; (2) LR-5 on both CEUS and CT/MRI with elevated carbohydrate antigen 19-9 (CA19-9); (3) LR-M on both CEUS and CT/MRI with elevated alphafetoprotein (AFP). The sensitivity, specificity, accuracy and area under the receiver operating characteristic curve (AUC) were calculated.

**Results:**

The rates of APHE and Rim-APHE on CEUS in cHCC-CCAs were 81.5% and 9.3%, respectively. The rate of early and marked washout on CEUS in cHCC-CCAs were 59.3% and 27.8%, respectively. 64.8% and 25.9% of cHCC-CCAs showed APHE and Rim-APHE on CT/MRI, respectively. 46.3% and 35.2% of cHCC-CCAs showed washout and delay enhancement on CT/MRI, respectively. The kappa value of LI-RADS categories of cHCC-CCAs on CEUS and CT/MRI was 0.319 (*P*=0.008). The sensitivity, specificity, accuracy and AUC of the aforementioned diagnostic criteria for cHCC-CCAs were 64.8%, 84.4%, 76.1% and 0.746, respectively.

**Conclusion:**

The combination of the CEUS and CT/MRI LI-RADS with serum tumor markers shows promising diagnostic performance of cHCC-CCAs.

## Introduction

Combined Hepatocellular-cholangiocarcinoma (cHCC-CCAs) comprise a minority (2.0%-5.0%) of primary hepatic malignancies (1). Tumors with both unambiguously differentiated hepatocellular and biliary components are defined as cHCC-CCAs, based on the 2019 World Health Organization classification (1). The origin, biological behavior, treatment method and prognosis of cHCC-CCAs differ from HCCs and intrahepatic cholangiocarcinoma (ICCs), the first and second common primary hepatic malignancies (2, 3). Liver resection may be the optimal treatment method for cHCC-CCAs, as pointed out by recent studies (4, 5). Thus, accurate diagnosis of cHCC-CCAs is of great importance in selecting treatment methods and performing patient management. In the past ten years, the pre-treatment diagnosis of cHCC-CCAs may sometimes be ignored by clinicians due to its low probability. Recently, knowledge for this specific type of tumor accumulates through clinical practice and is widely reported, which makes the pre-treatment diagnosis of cHCC-CCAs by contrast-enhanced imaging modalities a frontier of medical imaging (6–10).

cHCC-CCAs show various imaging features similar to HCCs and ICCs, which makes the differential diagnosis between them challenging. Recently, combining contrast-enhanced imaging and biomarkers to diagnose cHCC-CCAs shows promising potential for differentiating cHCC-CCAs from HCCs and ICCs (8, 11, 12). However, the diagnostic performance of mono-modality contrast-enhanced imaging with biomarkers for cHCC-CCAs is still unsatisfactory. As recently reported, the sensitivities for cHCC-CCAs reported in two studies (11, 12) were 32.5% and 50%, respectively, far from meeting clinical requirements.

In order to standardize the enhanced imaging for focal liver lesions, The American College of Radiology (ACR) published LI-RADS for CT/MRI and CEUS (13, 14). LI-RADS classifies liver lesions based on the size and imaging features and provides corresponding clinical management strategies. Compared with traditional enhanced imaging diagnosis, LI-RADS defines the image features and classifies lesions more definitely and detailly. The LR-M category of LI-RADS aims to differentiate HCCs from other non-HCC malignancies, which may be used as a reference for the diagnosis of cHCC-CCAs.

Previously, either enhancement patterns or LI-RADS combined with biomarkers were used as diagnostic criteria for cHCC-CCAs (11, 12). Usually, mono-modality was included in the criteria. The combination of multi-modality imaging methods in the differential diagnosis of cHCC-CCAs has not been mentioned before. We notice that ICCs may demonstrate different enhancement patterns on CEUS and CT/MRI due to the biliary components and their different principles of enhanced imaging modalities (15, 16). Inspired by the aforementioned facts, we infer in this study that cHCC-CCAs can also show inconsistent enhancement patterns and be classified into different LI-RADS categories on CEUS and CT/MRI, which may provide a practically useful way for the diagnosis of cHCC-CCAs.

Therefore, we aim to combine the CEUS and CT/MRI LI-RADS with tumor biomarkers to differentiate cHCC-CCAs from HCCs and investigate the diagnostic efficacy of the new criteria.

## Materials and Methods

The study was approved by the research ethics board. Pathologically confirmed cHCC-CCAs in two centers between 2013 and 2021 were retrospectively collected in this study. Inclusion criteria were (1) patients with pathologically confirmed cHCC-CCAs, (2) patients with high risk for HCCs, (3) patients with pre-treatment CEUS and contrast enhanced CT/MRI within 1 month, and (4) patients with the examination of alphafetoprotein (AFP) and carbohydrate antigen 19-9 (CA19-9) levels before treatment. We randomly selected HCCs in the same period as the time of cHCC-CCAs collection to satisfy a 1:1 proportion. A total of 54 cHCC-CCAs and 55 HCCs patients were collected.

### CEUS Examination

Patients underwent B-mode ultrasound and CEUS examination by an ultrasound system, such as EPICQ 7 (Philips Medical Solutions) and SIEMENS 3000 (Siemens Healthineers), equipped with an abdominal convex transducer (frequency range of 2.0-5.0 MHz). For the CEUS examination, 1.2 to 2.0 mL contrast agent (SonoVue, Bracco) was injected intravenously and flushed with 5 mL of 0.9% saline solution. The imaging timer was started immediately upon completion of injection. The target lesion was observed for 4 to 6 minutes and then the images was stored.

### Contrast-Enhanced CT/MRI Examinations

Dynamic contrast enhanced CT scanning was performed by Somatom Definition Flash dual-energy CT (Siemens Medical Solutions). The contrast agent, Iohexol (350mgl/ml, Beilu Pharmaceutical Co., Ltd) at a dosage of 1.2 ml/kg body weight and a flow rate of 3.5 ml/s, was injected with a pressure injector *via* the median cubital vein. The hepatic arterial phase imaging acquisition started at about 25 s to 35 s after the initiation of contrast injection. The portal venous phase imaging acquisition started at about 50 s to 70 s after the initiation of contrast injection, and the late phase was at about 180 s after the initiation of contrast injection.

Contrast-enhanced MRI scans were performed according to each institution’s protocol for focal liver lesions. MR imaging was performed with Siemens Magnetom Verio 3.0T magnetic resonance unit (Siemens Medical Solutions). Liver MR imaging protocol consisted of in-phase and opposed-phase T1 weighted imaging, FSE T2-weighted imaging with fat suppression and diffused weighted imaging. Gadoxetic acid (Primovist; Bayer Healthcare) was used as the contrast agent for EOB-DTPA enhanced imaging (EOB-MRI). Ultravist was used as the contrast agent for extracellular contrast agent MRI (ECA-MRI). Arterial, portal venous and delay (or transitional) phase images were acquired at delay times of 15 s to 18 s, 50 s to 60 s and 180 s after contrast injection using Volumetric Interpolated Breath-hold Examination (VIBE) sequence (TR/TE/FA, 4.2/1.5/9, 300×400 matrix). For EOB-MRI, Hepatobiliary phase imaging was completed 20 minutes after the contrast injection.

### Image Analysis

All observers were blinded to pathology and other imaging results. One observer (J. D. with more than 12 years of experience in liver CEUS) reviewed the CEUS images of liver nodules and assigned a category to each nodule based on CEUS LI-RADS (2017 version) (14). The observers determined the presence or absence of the following features based on definitions proposed by CEUS LI-RADS (2017 version): (1) size, (2) arterial phase hyperenhancement (APHE), (3) mild or late washout, (4) ancillary features, including definite growth, nodule-in-nodule and mosaic architecture. The criterion for CEUS LR-M was lesions with Rim-APHE or early washout or marked washout. One radiologist (X. Z. with more than 15 years of experience in CT/MRI) reviewed the CT/MRI images of lesions and classified the lesion into the corresponding category based on CT/MRI LI-RADS (2018 version) (13). The observers determined the presence or absence of the following features based on definitions proposed by CT/MRI LI-RADS (2018 version): (1) size, (2) APHE, (3) washout appearance according to the type of MRI (conventional washout was defined as hypointensity on the PVP or DP on ECA-MRI or hypointensity on the PVP on EOB-MRI), (4) enhancing “capsule”, (5) threshold growth, (6)ancillary features, including restricted diffusion, mild-moderate T2 hyperintensity, corona enhancement, transitional phase hypointensity, hepatobiliary phase hypointensity, nodule-in-nodule and mosaic architecture. According to the diagnostic algorithm of ACR LI-RADS, lesions in LR-1 are defined as definitely benign lesions, LR-2, benign lesions, LR-3, the intermediate probability of malignancies, LR-4, probably HCCs, LR-5, definitely HCCs, LR-TIV, definite tumors in vein and LR-M, probably or definitely but not HCC-specific malignancies. Some uncommon HCCs and most of the non-HCC malignancies can be classified into the LR-M. Thus, LR-M can differentiate HCCs from other malignancies.

The diagnostic criteria for cHCC-CCAs if one or more of the following conditions were satisfied: (1) APHE on CEUS and CT/MRI LR-M; (2) CEUS LR-5 and CT/MRI LR-5 with elevated CA19-9; (3) CEUS LR-M and CT/MRI LR-M with elevated AFP.

### Statistical Analysis

Quantitative data were expressed as the mean ± standard deviation. Qualitative data were presented as numbers and percentages. Differences in quantitative variables were tested by the independent sample t-test. Comparison of the categorical variables was performed by the χ^2^ test or Fisher’s. CEUS and CT/MRI LI-RADS for each lesion was assessed by Cohen’s kappa. The area under the receiver operating characteristic curve (AUC) was used to analyze the performance of the diagnostic criteria. The sensitivity, specificity, accuracy, positive predictive value (PPV) and negative predictive value (NPV) for cHCC-CCAs were calculated. A *P* value < 0.05 indicated a significant difference. Statistical analyses were performed using the SPSS software, version 22.0 (SPSS Inc.,).

## Results

### Clinical Data of Patients

A total of 54 patients with cHCC-CCAs and 55 ones with HCCs were included in this study. No significant differences in sex, age, etiology, tumor size and tumor markers were observed between the two groups (*P* > 0.05). There were more patients with liver cirrhosis in the HCCs group than that in the cHCC-CCAs group. In addition, the percentage of patients in the HCCs group undergoing ultrasound-guided biopsy was higher than that for the cHCC-CCAs group. The clinical characteristics of patients in the two groups were shown in **Table 1**. In the group of cHCC-CCAs, 15 patients underwent ECA-MRI and 11 patients underwent EOB-MRI. In the group of HCCs, only one patient underwent MRI with extracellular agents and 29 patients underwent EOB-MRI.

**Table 1:** Clinical characteristics of patients in the cHCC-CCAs and HCCs groups.

	cHCC-CCAs	HCCs	*P*
Sex (Male/Female)	43/11	45/10	0.772
Age	58.7 ± 9.6	57.2 ± 9.9	0.406
Etiology (Hepatitis B virus/Hepatitis C virus/Others)	44/4/6	46/3/6	0.915
Liver cirrhosis (Yes/No)	39/15	49/6	0.026
Tumor size on CEUS (cm)	4.42 ± 2.49	3.95 ± 2.23	0.306
Tumor size on CT/MRI (cm)	4.37 ± 2.46	3.72 ± 2.17	0.149
Pathological specimen (liver resection/ultrasound guided biopsy)	44/10	28/27	0.001
AFP (>15ng/ml/≤15 ng/ml)	35/19	31/24	0.367
CA199 (>39ng/ml/≤39 ng/ml)	19/35	13/42	0.186

### Imaging Features of cHCC-CCAs and HCCs on CEUS and CT/MRI

A total of 81.5%, 9.3% and 9.3% lesions in the cHCC-CCAs group and 94.5%, 1.8% and 3.6% lesions in the HCCs group showed APHE, Rim-APHE and non-APHE in the arterial phase (χ^2 =^ 4.610, *P*=0.1), respectively. Early washout, marked washout, delay and mild washout and non-washout were observed in 59.3%, 27.8%, 37.0% and 1.9% of cHCC-CCAs, and 14.5%, 7.3%, 72.7% and 7.3% of HCCs, respectively. Early washout and marked washout were more frequent in cHCC-CCAs than that of HCCs (χ^2 =^ 28.339, *P*<0.001) (**Table 2**).

**Table 2:** Contrast enhancement patterns of cHCC-CCAs and HCCs on CEUS.

	cHCC-CCAs	HCCs
Arterial phase		
-APHE	44	52
-Rim APHE	5	1
-Non APHE	5	2
Portal and delay phases		
-Early washout	32	8
-Marked washout	15	4
-Delay and mild washout	20	40
-No washout	1	4

64.8%, 25.9% and 9.3% of cHCC-CCAs showed APHE, Rim-APHE, and non-APHE in the arterial phase, while the percentages of HCCs with these imaging features were 94.5%, 0, and 5.5%, respectively (χ^2 =^ 17.814, *P*<0.001). Hypo-enhancement, delayed enhancement and iso- or hyper-enhancement in the portal and delay phases of CT/MRI were observed in 46.3%, 35.2% and 18.5% of cHCC-CCAs, and 96.4%, 0 and 3.6% of HCCs, respectively. Statistical significance of image features in the portal and delay phases was observed between the two groups (χ^2 =^ 34.378, *P*<0.001) (**Table 3**)

**Table 3:** Contrast enhancement patterns of cHCC-CCAs and HCCs on CT/MRI.

	cHCC-CCAs	HCCs
Arterial Phase		
-APHE	35	52
-Rim APHE	14	0
-Non APHE	5	3
Portal and delay phases		
Hypo-enhancement	25	53
Delayed enhancement	19	0
Iso- or hyper enhancement	10	2

### LI-RADS Categorizations of cHCC-CCAs on CEUS and CT/MRI

5.6%, 37.0% and 57.4% of cHCC-CCAs were categorized to LR-4, LR-5 and LR-M by CEUS, while 3.7%, 35.2% and 61.1% of cHCC-CCAs were categorized to LR-4, LR-5 and LR-M by CT/MRI, respectively. The Kappa value of the intermodality classifications on CEUS and CT/MRI LI-RADS for cHCC-CCAs was 0.319, *P*=0.008 (**Table 4**)

**Table 4:** LI-RADS categorizations of cHCC-CCAs by CEUS and CT/MRI.

CT/MRI	CEUS			Total
	LR-4	LR-5	LR-M	
LR-4	0	1	2	3
LR-5	2	11	7	20
LR-M	0	7	24	31
Total	2	19	33	54

For HCCs, 1.8%, 9.1%, 69.1% and 20% lesions on CEUS, 1.8%, 16.4%, 81.8% and 0 on CT/MRI were categorized to LR-3, LR-4, LR-5 and LR-M, respectively. The Kappa value of the intermodality classifications on CEUS and CT/MRI LI-RADS for HCCs was 0.003 (*P*=0.968) (**Table 5**)

**Table 5:** LI-RADS categorizations of HCCs by CEUS and CT/MRI.

CT/MRI	CEUS				Total
	LR-3	LR-4	LR-5	LR-M	
LR-3	0	0	1	0	1
LR-4	0	1	6	2	9
LR-5	1	4	31	9	44
LR-M	0	0	0	0	0
Total	1	5	38	11	55

### Diagnostic Performance of CEUS and CT/MRI LI-RADS in Association With Serum Biomarkers for the Diagnosis of cHCC-CCAs

**Figure 1:**
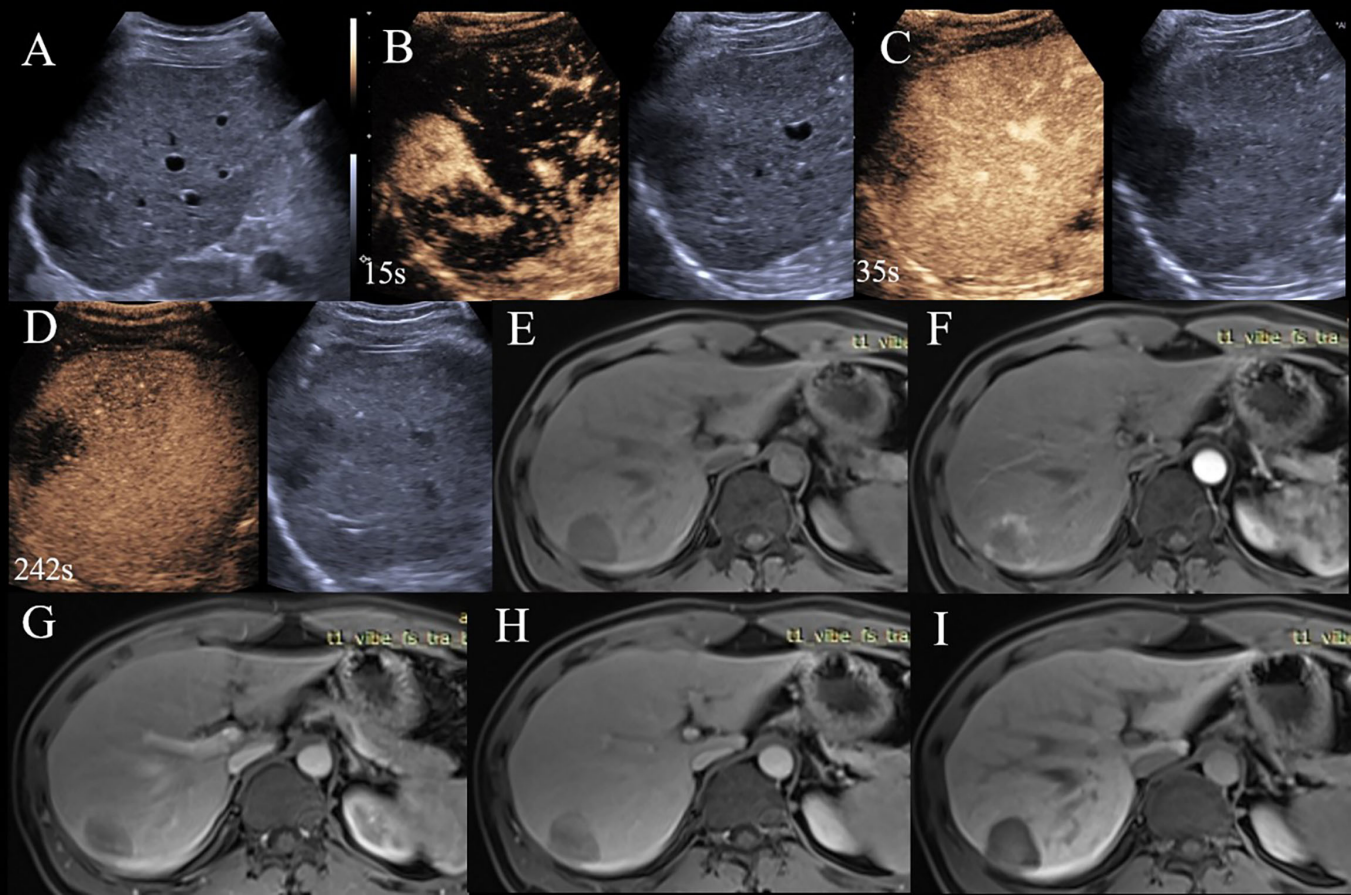
A 36-year-old man with HBV related liver cirrhosis. Serologic data indicated AFP of 167 ng/ml and CA19-9 of 13.5 ng/ml. **(A)** A hypo echoic lesion located under the liver capsule with the size of 4.1×3.8cm. **(B)** The lesion displayed APHE on CEUE; **(C)** Early washout was observed at 35s after injection of contrast agent; **(D)** Washout was observed on delay phase, the lesion was categorized as CEUS LR-M. **(E)** The lesion displayed hypointensity on EOB-MRI; **(F)** Rim-APHE was observed on EOB-MRI; **(G, H)** Delayed enhancement was also observed on portal and transitional phases; **(I)** The lesion showed hypointensity on hepatobiliary phase, the lesion was EOB MRI LR-M. The final diagnosis was cHCC-CCA.

**Figure 2:**
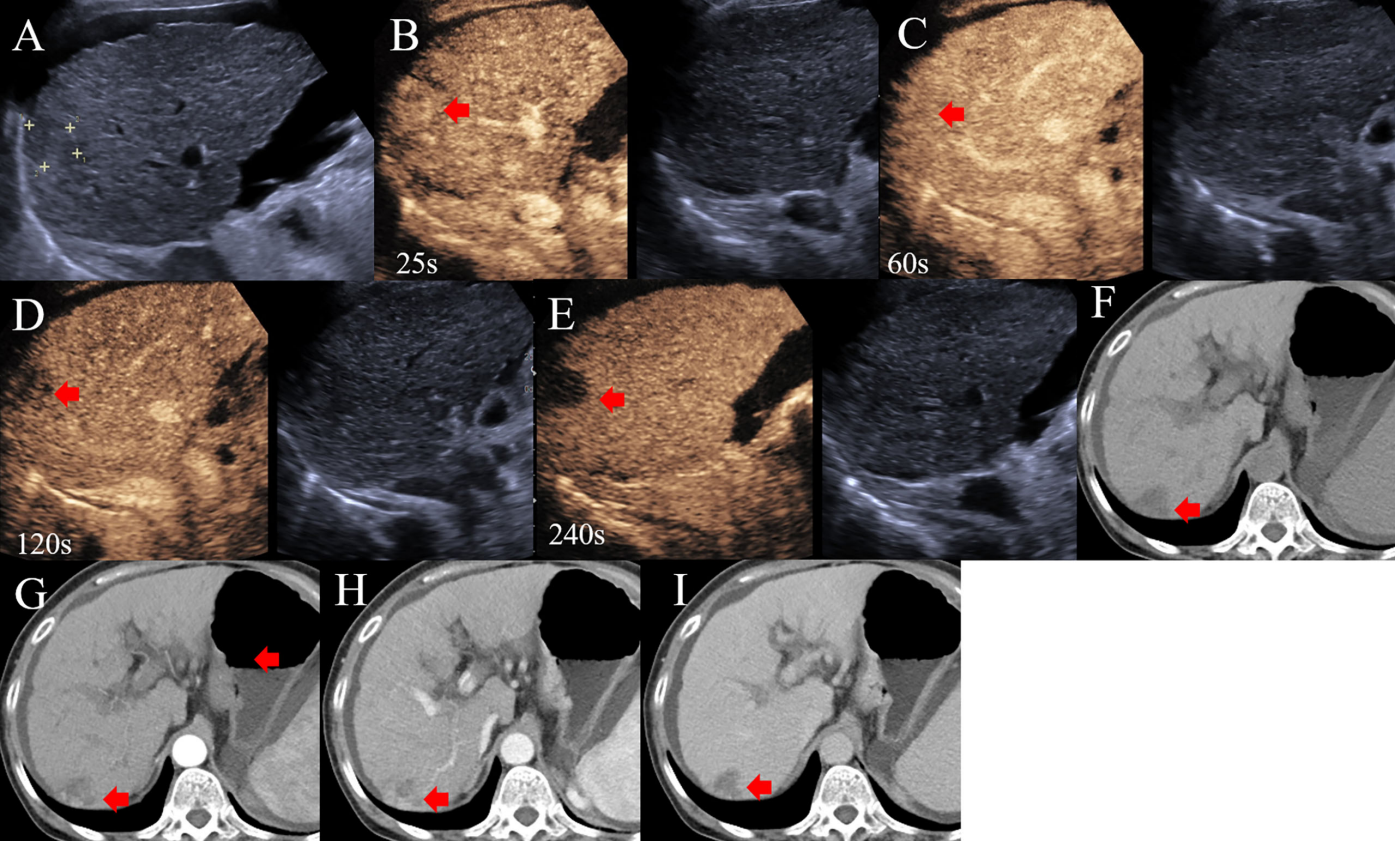
A 68-year-old woman with HBV related liver cirrhosis and elevated AFP (1210ng/ml) and CA19-9 (43.03 ng/ml). **(A)** A hypo-echoic lesion with a size of 1.9cm was observed by US. **(B)** The lesion showed APHE on CEUS; **(C)** without washout 1 min after injection of contrast agent; **(D)** Delay and mild washout was observed 2 min after injection; **(E)** The lesion appeared punched-out 4 min after injection of contrast agent. The lesion was categorized to CEUS LR-5. **(F)** A hypo-intensive lesion was found on CT; **(G, H)** The heterogeneous enhancement was observed on the arterial and portal phases of contrast enhanced CT; **(I)** The lesion was hypo-intensive on the delay phase and classified into CT LR-5. The final diagnosis was cHCC-CCAs, as confirmed by pathology.

**Figure 3:**
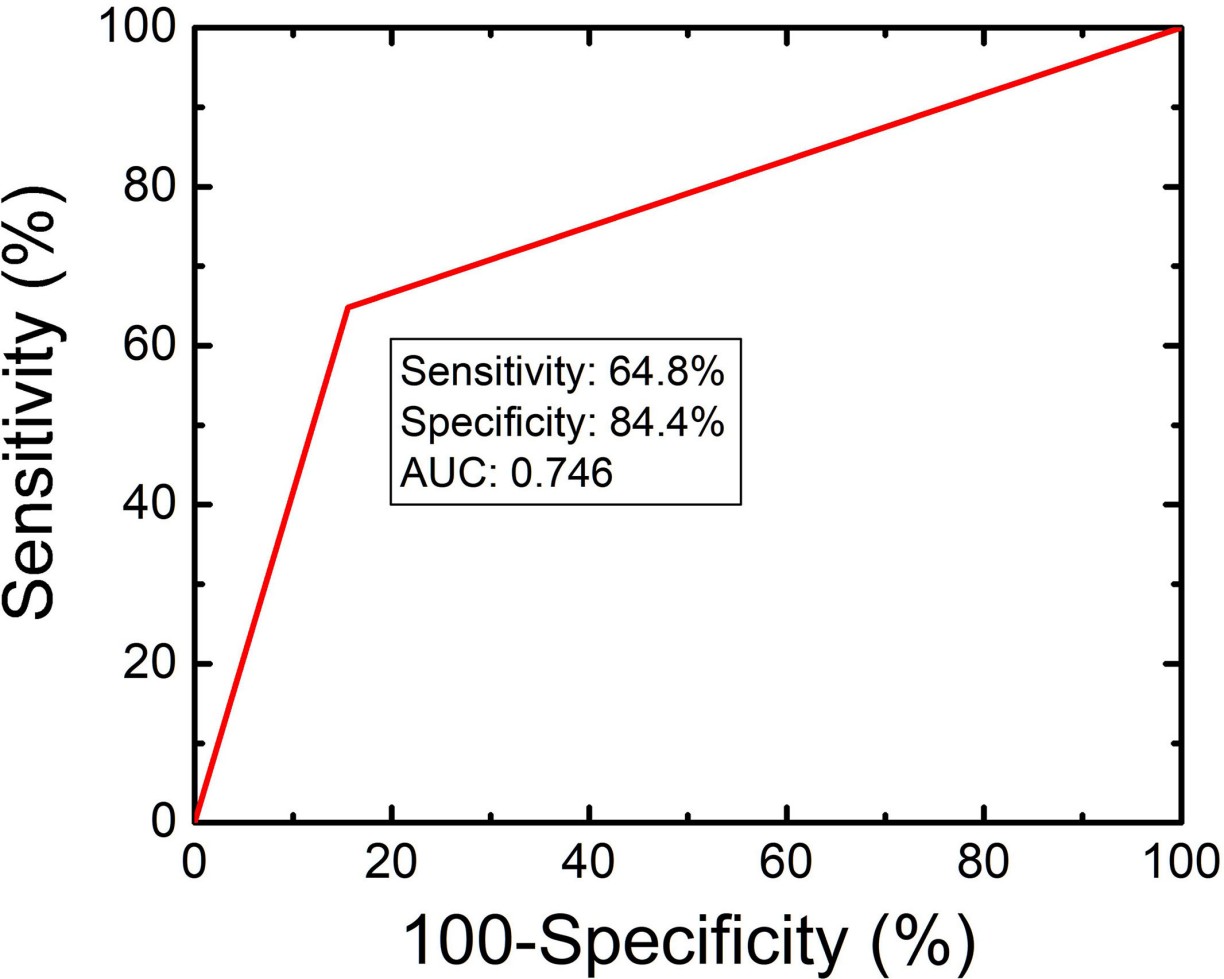
Diagnostic performance of CEUS and CT/MRI LI-RADS in association with serum biomarkers for the diagnosis of cHCC-CCAs.

We provided three diagnostic criteria, mentioned above, for cHCC-CCAs from HCCs. 35 cHCC-CCAs and 7 HCCs met at least one of the three criteria mentioned above (**Figures 1, 2**). 25 cHCC-CCAs showed APHE on CEUS and were in CT/MRI LR-M; (2) 6 cHCC-CCAs were in CEUS LR-5 and CT/MRI LR-5 with elevated CA19-9; (3) 15 cHCC-CCAs were in CEUS LR-M and CT/MRI LR-M with elevated AFP. 7 HCCs were in CEUS LR-5 and CT/MRI LR-5 with elevated CA19-9.The sensitivity, specificity, accuracy, PPV and NPV of the criteria for the diagnosis of cHCC-CCAs were 64.8%, 84.4%, 76.1%, 87.6% and 71.6%, respectively. The AUC was 0.746. (**Figure 3**).

## Discussion

cHCC-CCAs is a subtype of primary liver cancer with a low incidence compared with HCCs and ICCs (17). cHCC-CCAs can present imaging features similar to both HCCs and ICCs, which makes its differential diagnosis challenging. In this study, we show that CEUS and CT/MRI LI-RADS, presented by ACR, in association with serum biomarkers for differentiating cHCC-CCAs from HCCs, has significant diagnostic performance and can provide a diagnostic reference in clinical practice.

HCCs and ICCs are easy to diagnose based on the typical enhancement patterns on CT/MRI and CEUS (18, 19). However, the enhancement patterns of cHCC-CCAs are affected by the proportions of HCC- or ICC-like histologic components, leading to a significant barrier for the diagnosis of cHCC-CCAs (20). Recently, several studies focused on the combination of contrast-enhanced patterns and serum biomarkers to diagnose cHCC-CCAs due to the lack of typical enhanced patterns (8, 11, 12). The diagnostic criteria for cHCC-CCAs mentioned by Li et al. (11) and Huang et al. (12) include lesions with typical imaging features of HCCs and elevated CA19-9, lesions with typical imaging features of ICCs and elevated AFP, and lesions with typical imaging features of HCCs or ICCs with both elevated CA19-9 and AFP. Li et al. (11) showed a promising sensitivity in the diagnosis of cHCC-CCAs when using as the diagnostic criterion the simultaneous elevation of AFP and CA19-9, or different diagnostic results from tumor markers and CEUS (51.1%), and contrast-enhanced CT (53.5%). These results indicated that almost half of cHCC-CCAs were misdiagnosed even if the combination of imaging features and tumor markers were adopted as the diagnostic criteria.

ACR developed CT/MRI and CEUS LI-RADS to standardize categorization for liver lesions in high-risk patients and improve communication of clinicians by classifying the lesions into LR-1 to LR-5, LR-M and LR-TIV. Among the seven classes, LR-5 shows a high PPV and specificity for HCCs, which provides a reference for physicians in clinical decision-making (13, 14). Almost all the previous studies reported that the PPVs of both CEUS and CT/MRI LR-5 for HCCs were above 95% (21–23). Thus, CEUS and CT/MRI LR-5 can be used as diagnostic criteria for HCCs. Therefore, LI-RADS provides a possibility for the differential diagnosis of cHCC-CCAs and HCCs (24–26). Using either CEUS LR-5 with elevated CA19-9, CEUS LR-M with elevated AFP or CEUS LR-5/LR-M with both elevated CA19-9 and AFP, as the diagnostic criteria for cHCC-CCAs, the AUC, sensitivity and specificity were 0.649, 40.0% and 89.9%, respectively (27). This result preliminarily demonstrated the diagnostic value of LI-RADS combined with tumor markers for cHCC-CCAs.

Although imaging features and elevated tumor markers attract attention in the diagnosis of cHCC-CCAs, the possible indication of the discordance between contrast-enhanced patterns in CEUS and CT/MRI was ignored. ICCs, which show “wash-in and washout” on CEUS and “Rim-APHE and delayed enhancement” on CT/MRI, have inconsistent contrast enhanced patterns in CEUS and CECT/MRI due to the different imaging principles. This discordance between contrast-enhanced patterns provided critical imaging information in the diagnosis of ICCs (15, 16). cHCC-CCAs have the same histologic components as ICCs. We, therefore, hypothesize that the discordance between contrast-enhanced patterns of CEUS and CT/MRI may be an indication for cHCC-CCAs.

In our study, we compared the major imaging features of cHCC-CCAs and HCCs on CEUS and CT/MRI. The results reveal that most cHCC-CCAs and HCCs showed APHE on CEUS without a statistical significance. The frequencies of marked washout and early washout in cHCC-CCAs, however, were higher than those of HCCs, which were consistent with a previous study (27). On CT/MRI, the frequencies of Rim-APHE and delayed enhancement in cHCC-CCAs were higher than those of HCCs, respectively. For the LI-RADS categorization, most of cHCC-CCAs were classified to CT/MRI and CEUS LR-5 or LR-M. The Kappa value of the inter-modality of the classifications by CEUS and CT/MRI LI-RADS for cHCC-CCAs was 0.319, indicative of a significant inconsistency between the two imaging methods. Most of the HCCs, on the contrary, were categorized to LR-5 both in CEUS and CT/MRI LI-RADS.

We propose new diagnostic criteria for cHCC-CCAs. as mentioned above, based on the combination of the different diagnostic results from the enhancement pattern on CEUS and CT/MRI and tumor markers. The result suggests that our new diagnostic criteria have a good performance for cHCC-CCAs. Yang et al. (27) presented that “CEUS LR-M with elevated AFP” can be one of the diagnostic criteria for cHCC-CCAs. However, several studies found that 50% to 75% of lesions in CEUS LR-M were HCCs (23, 28), which was usually accompanied by an elevation of AFP. Thus, it can be inferred that the criterion “CEUS LR-M with elevated AFP” may lead to misdiagnosis. In the present study, we used the “CEUS LR-M and CT/MRI LR-M with elevated AFP” instead of “CEUS LR-M with elevated AFP” as a diagnostic criterion. Our choice is based on the fact that few HCCs can be categorized as both CT/MRI and CEUS LR-M.

There are a few limitations of our study. First, we included HCCs but not ICCs in the control group. Second, the inter-reader agreement between CEUS and CT/MRI LI-RADS was not explored.

In conclusion, most of cHCC-CCAs were categorized to LR-5 and LR-M by both CEUS and CT/MRI LI-RADS. By combining CEUS and CT/MRI LI-RADS in association with serum biomarkers we presented new criteria for the diagnosis of cHCC-CCAs. The results show that the new diagnostic algorithm shows a prior diagnostic performance. We believe the diagnostic criteria shown in this study can be used to help clinical decision-making.

## Data Availability Statement

The raw data supporting the conclusions of this article will be made available by the authors, without undue reservation.

## Author Contributions

YZ and SY designed the study and wrote the manuscript. YZ, SY, LZ, XZ, ML, and JD collected the data. KY and XJ supervised the findings of this study. All authors contributed to the article and approved the submitted version.

## Funding

The present work was supported by Tianjin health and Health Committee (No. MS20017, KJ20170, ZD20014, NQ20033) and founded by Tianjin Key Medical Discipline (Specialty) Construction Project.

## Conflict of Interest

The authors declare that the research was conducted in the absence of any commercial or financial relationships that could be construed as a potential conflict of interest.

## Publisher’s Note

All claims expressed in this article are solely those of the authors and do not necessarily represent those of their affiliated organizations, or those of the publisher, the editors and the reviewers. Any product that may be evaluated in this article, or claim that may be made by its manufacturer, is not guaranteed or endorsed by the publisher.
